# Childhood chronic urticaria and type 1 diabetes

**DOI:** 10.1186/1710-1492-7-S2-A18

**Published:** 2011-11-14

**Authors:** A Mazzetti, R Borici-Mazi

**Affiliations:** 1 Third year medical student, Queens University, Kingston, Ontario, Canada; 2 Division of Allergy and Immunology, Queens University, Kingston, Ontario, Canada

## Background

Chronic spontaneous urticaria is a common condition encountered in childhood, but long lasting persistent urticaria is less frequent. Autoimmune mechanisms may explain up to 30-50% of chronic idiopathic urticaria in adults, but such etiology has been less studied in children.

## Method

Case report and literature review.

## Results

We present the case of a 10 year old, otherwise healthy boy, who has a 6-7 year history of persistent chronic urticaria requiring ongoing treatment. His symptoms consist of daily urticarial lesions and angioedema with frequent flares. Upper respiratory viral infections, spring/ fall season and stressful events make his condition worse; however, no food or medication triggers has been found so far. The initial work up of chronic urticaria included CBC, differential, TSH, thyroid antibodies, ANA, C3 and C4 titers, serology for H. Pylori, tryptase level, ESR, stool for O&A, urinalysis, which were entirely negative. A year ago, he developed sudden onset of weight loss, polydipsia and polyuria, and was diagnosed with Type 1 insulin dependent diabetes. He was found to have positive antiGAD antibodies, but thyroid and anti TTG IgA antibodies remained negative. He has been tried on many treatment modalities including various combinations of new and old generation antihistamines, steroids, ketotifen, montelukast, but his condition remains active.

## Conclusion

As extension to current guideline for work up of chronic urticaria, besides thyroid and high affinity anti FcεRI receptor autoantibodies, screening for anti GAD and antiTTG IgA antibodies can be considered in cases of persistent long lasting chronic urticaria in childhood.

